# Chronic stability of activated iridium oxide film voltage transients from wireless floating microelectrode arrays

**DOI:** 10.3389/fnins.2022.876032

**Published:** 2022-08-08

**Authors:** Rebecca A. Frederick, Ellen Shih, Vernon L. Towle, Alexandra Joshi-Imre, Philip R. Troyk, Stuart F. Cogan

**Affiliations:** ^1^Neural Interfaces Laboratory, Department of Bioengineering, The University of Texas at Dallas, Richardson, TX, United States; ^2^Clinical Neurophysiologic Mapping Laboratory, Department of Neurology, The University of Chicago, Chicago, IL, United States; ^3^Laboratory of Neuroprosthetic Research, Department of Biomedical Engineering, Illinois Institute of Technology, Chicago, IL, United States

**Keywords:** wireless, neural stimulation, AIROF, material stability, microelectrode array (MEA)

## Abstract

Successful monitoring of the condition of stimulation electrodes is critical for maintaining chronic device performance for neural stimulation. As part of pre-clinical safety testing in preparation for a visual prostheses clinical trial, we evaluated the stability of the implantable devices and stimulation electrodes using a combination of current pulsing in saline and in canine visual cortex. Specifically, in saline we monitored the stability and performance of 3000 μm^2^ geometric surface area activated iridium oxide film (AIROF) electrodes within a wireless floating microelectrode array (WFMA) by measuring the voltage transient (VT) response through reverse telemetry. Eight WFMAs were assessed *in vitro* for 24 days, where *n* = 4 were pulsed continuously at 80 μA (16 nC/phase) and *n* = 4 remained in solution with no applied stimulation. Subsequently, twelve different WFMAs were implanted in visual cortex in *n* = 3 canine subjects (4 WFMAs each). After a 2-week recovery period, half of the electrodes in each of the twelve devices were pulsed continuously for 24 h at either 20, 40, 63, or 80 μA (200 μs pulse width, 100 Hz). VTs were recorded to track changes in the electrodes at set time intervals in both the saline and *in vivo* study. The VT response of AIROF electrodes remained stable during pulsing in saline over 24 days. Electrode polarization and driving voltage changed by less than 200 mV on average. The AIROF electrodes also maintained consistent performance, overall, during 24 h of pulsing *in vivo*. Four of the *in vivo* WFMA devices showed a change in polarization, access voltage, or driving voltage over time. However, no VT recordings indicated electrode failure, and the same trend was typically seen in both pulsed and unpulsed electrodes within the same device. Overall, accelerated stimulation testing in saline and *in vivo* indicated that AIROF electrodes in the WFMA were able to consistently deliver up to 16 nC per pulse and would be suitable for chronic clinical use.

## Introduction

Implementing wireless communication for implanted neural interfaces can be beneficial for reducing the number of implanted components and minimizing the risk of complications related to the use of cables and connectors, including infection at percutaneous connectors, electrode migration, lead fracture, and exacerbation of the foreign body response due to tethering forces. Power supply for wireless neural interfaces is most often accomplished with near-field magnetic resonant coupling (Samineni et al., 2017; Gutruf et al., 2018; Won et al., 2021). Recent developments in power delivery systems include the use of piezoelectric components combined with ultrasound (Seo et al., 2016), photovoltaics with optical excitation (Lee et al., 2018), and harvesting methods utilizing kinetic energy (Hwang et al., 2015; Yao et al., 2018), chemical energy (Rapoport et al., 2012; Falk et al., 2013), or thermal energy (Settaluri et al., 2012; Nan et al., 2018) from the body to generate power. A major benefit of magnetic resonant coupling is that it facilitates both wireless power transfer and wireless communication between implanted and external system components, while other power delivery methods require additional components or systems (e.g., Bluetooth) for wireless communication (Rapoport et al., 2012; Park et al., 2015; Seo et al., 2015, 2016; Mickle et al., 2019).

Most wireless communication systems for neural interfaces have focused on either providing stimulation waveforms for neural excitation or recording neural electrical activity (Harrison et al., 2008; Yin et al., 2014; Luan et al., 2020; Mcglynn et al., 2021; Gilron et al., 2021), and few have provided a means to monitor the integrity or performance of the electrodes. For stimulating electrodes, clinical implanted pulse generators are typically able to report a single-point impedance during current or voltage pulsing. Some implanted pulse generators, primarily in cardiac applications, can also record and store electrical activity for later upload via a wireless link. None of these approaches provide data on the electrode potential during an applied stimulation pulse.

Knowledge of the electrode’s potential, or the voltage transient (VT) response, during stimulation provides information on whether the applied stimulation may generate tissue damage by catalyzing reactions with harmful products, or possibly cause damage to the electrode itself. In wired systems, implanted electrodes can be monitored by a variety of electrochemical measurements including: impedance spectroscopy, cyclic voltammetry, or recording the voltage transient response to stimulation current pulsing. However, these methods for monitoring an electrode (and stability of the electrode-tissue interface) may be difficult to implement in battery-less wireless systems. There are a number of technical challenges in implementing electrochemical testing in wireless neural interfaces. First, limited power supply may limit the voltage (or current) range over which testing can be performed. Second, data sampling rates required to capture some electrochemical measurements may be limited in wireless systems. Third, most neural stimulation devices are designed with circuits and system components for constant current supply while neural recording devices are designed with circuits for voltage recording and signal processing. The most common electrochemical tests for neural interfaces require ramped (CV) or sinusoidal (EIS) voltage supply with current monitoring, or current supply (VT) with voltage monitoring and control. Although all these capabilities can be included within the same system, it would likely increase the overall size of the implanted device to be inappropriate for many neural interface applications. The requirements for implementing VT measurements are most similar to existing wireless neural stimulator designs, and are therefore the easiest to implement without significantly increasing the size of implanted components. Additional focus on the development of strategies for wireless systems to capture electrode voltages during constant-current stimulation will be particularly beneficial for translating wireless neural interfaces to successful long-term clinical use. Where tracking the VT response can help ensure the safety of neural stimulation and stability of electrodes and electrode coatings during chronic studies.

Stimulation charge-injection with iridium oxide electrodes is accompanied by the ingress or outflow of charge-balancing counter ions and changes in redox-oxidation state of the oxide, between at least Ir^3+^ and Ir^4+^ valence states. The electrochemical potential range over which the oxide is driven and the accompanying volume changes in the iridium oxide film may drive irreversible reactions that degrade either the electrode or adjacent tissue. Activated iridium oxide film (AIROF) has a particularly low density for an iridium oxide, making the electrode vulnerable to physical disruption and alteration of properties by adsorption of biomolecules onto or into the three-dimensional structure of the oxide. Therefore, it is essential to establish the stability of AIROF stimulation electrodes at the maximum anticipated levels of clinical charge-injection, particularly for implanted AIROF electrodes.

Based on studies by Schmidt et al. (1996), intracortical microstimulation of the visual cortex in a non-sighted human volunteer showed thresholds of 0.4–4.5 nC/phase might be expected for eliciting phosphenes with cathodal-first stimulation pulses. The cathodal-first threshold charge per phase reported by Schmidt et al., corresponds to a charge density of 0.013–0.15 mC/cm^2^ for the 3000 μm^2^ AIROF electrodes on the wireless floating microelectrode array (WFMA). These charge densities are well below the maximum reversible charge densities reported for positively biased AIROF electrodes based on avoiding water electrolysis. It should be noted, however, that Schmidt et al. (1996), did not employ a positive interpulse bias and for thresholds requiring higher currents, greater than 30 μA, they employed anodal-first biphasic current pulses and reported phosphene thresholds of 8.8–15.4 nC/phase (44–77 μA at the 200 μs pulse duration employed in their study). These thresholds correspond to charge densities of 0.29–0.51 mC/cm^2^ for 3000 μm^2^, which remain within reported charge-injection limits for AIROF electrodes from saline studies but may exceed charge-injection limits based on acute *in vivo* studies (Hu et al., 2006). Whether cathodal-first thresholds at these charge levels will be required for AIROF electrodes pulsed from a positive interpulse bias remains to be determined.

Activated iridium oxide film (AIROF) microelectrodes were previously used for stimulation in animal studies in cortex (Agnew et al., 1986; McCreery et al., 1986; Anderson et al., 1989; Weiland and Anderson, 2000; Liu et al., 2006) and in spinal cord (McCreery et al., 2004), and in human clinical studies in visual cortex (Bak et al., 1990; Hambrecht, 1995; Schmidt et al., 1996). Prior work has shown evidence of tissue damage and film delamination when 2000 μm^2^ surface area AIROF electrodes were pulsed at charge densities of 3 mC/cm^2^ (60 nC/phase, 100 μA, 600 μs pulse width), but not when pulsed at 2 mC/cm^2^ (40 nC/phase, 100 μA, 400 μs pulse width) (Cogan et al., 2004). Additionally, AIROF formed from sputter-deposited iridium metal (rather than iridium microwires) showed evidence of delamination when pulsed at 50 nC per phase (0.5 mC/cm^2^), but not at 40 nC per phase (0.9 mC/cm^2^) (Negi et al., 2010).

No prior work has evaluated AIROF microelectrode stability when used in a wireless stimulation system. We have attempted to establish the stability of AIROF for clinical use in a wireless and battery-less intracortical vision prosthesis. Our investigation combined an aggressive, accelerated pulsing study in a buffered saline electrolyte with an accelerated, short term pulsing study of AIROF implanted in canine visual cortex. The maximum stimulus intensity of 16 nC/phase employed in the present study, for both saline and canine testing, is anticipated to encompass likely clinical levels of stimulation. Voltage transient measurements recorded during stimulation current pulsing and transmitted via reverse telemetry were used to track electrode stability over time. In this study, we first present data from a long-term pulsing study of WFMA devices in saline and then from a 24-h pulsing study of WFMA devices implanted in canine visual cortex. These data were collected as part of the pre-clinical safety assessment of an intracortical visual prosthesis system in which neural stimulation electrodes are implanted in the visual cortex and powered and controlled extracorporeally by a wireless transcutaneous link [NCT04634383].

## Materials and methods

### Device information

The intracortical visual prosthesis (ICVP) system consists of a group of WFMA devices, an external telemetry coil and telemetry control (TC) unit, and a custom software module. Each WFMA device is composed of a 16-channel microelectrode array with ceramic substrate, application-specific integrated circuit (ASIC), and power/telemetry coil. The microelectrode array is comprised of 16 AIROF stimulation electrodes and 2 uninsulated iridium electrode shafts used as counter and reference electrodes. For stimulation electrodes, iridium microwires are insulated with Parylene-C and excimer laser ablation is used to expose a geometric surface area (GSA) of 3000 μm^2^ at the tips. Electrodes are placed into the ceramic substrate and the ASIC is placed on the back of the substrate, where wire bonding is used to connect each electrode to contact pads on the ASIC. Activation of the electrodes is accomplished through the wireless system by potential cycling (100 cycles, 10 s hold at each step: +0.8 V and -0.6 V vs. iridium reference) with the device submerged in phosphate buffered saline.

The external TC unit contains a 4.5 MHz frequency-shift-keyed (FSK) class E converter for delivering power to the implanted WFMA and can receive data at 1.25 Mbps (Troyk and DeMichele, 2003). The ASIC within the WFMA uses the 4.5 MHz power carrier to derive the system clock. The system clock is then used to synchronously control all functions within the WFMA. The TC unit controls timing of the command stream to set the stimulation frequency and synchronization. For each command, the WFMA ASIC generates stimulation current using a compliance limited current delivery strategy with anodic bias (Cogan et al., 2006), and each electrode can be addressed independently. The maximum current output of the WFMA ASIC is 80 μA, due to the maximum wireless system power supply of approximately 3.5 V. And so, the maximum possible charge density for any electrode is 533 μC/cm^2^ at a 200 μs pulse width.

A data subcarrier at 140 kHz is used to transmit pulse-width modulated reverse telemetry back to the TC unit. The measured reverse telemetry signal is converted to voltage using a pulse-width to voltage conversion factor that is unique to each device. Data are recorded to a TXT file that reports the sample time, clock cycle count (as a measure of reverse-telemetry pulse width) for the stimulation electrode, clock cycle count for the reference electrode, voltage change per clock cycle (conversion factor for the device), and voltage offset. The voltage for the stimulation electrode and for the reference electrode are then calculated from the clock cycle counts using the conversion factor for the device. The voltages of the electrode with respect to the reference electrode values are the final values reported for the voltage transient measurement.

### Long-term pulsing in saline (24 days)

Saline pulsing studies were conducted at The University of Texas at Dallas. The stability of the AIROF electrodes within WFMA devices in response to long-term current pulsing was evaluated in buffered saline by concurrently measuring voltage transients and iridium dissolution. Each of eight WFMAs was placed inside a separate plastic collet that is part of a rapid-speed surgical tool system for aiding cortical implantation of the WFMAs. In the present study the collet served as a convenient holder for the WFMA to prevent mechanical damage during handling. The WFMAs were ethylene oxide sterilized prior to the pulsing study. WFMAs were assigned to either pulsed (*n* = 4) or unpulsed (*n* = 4) groups, and initial optical microscopy images were recorded for all devices in the pulsed group. Table 1 summarizes the grouping of devices into pulsed and unpulsed study groups and the total pulsing time and accumulated charge delivered by pulsed devices.

**TABLE 1:** WFMA IDs and group assignments for pulsing in saline.

Group	WFMA ID	Pulsing time (hours)	Total charge (Coulombs)
Pulsed	W0089_63_152	701.5	16.16
Pulsed	W0090_31_153	762.0	17.56
Pulsed	W0124_64_187	699.0	16.10
Pulsed	W0128_42_191	762.0	17.56
No Pulsing	W00019_156	0	0
No Pulsing	W00126_150	0	0
No Pulsing	W0132_101_196	0	0
No Pulsing	W0133_6_197	0	0

#### Test cells

**FIGURE 1:**
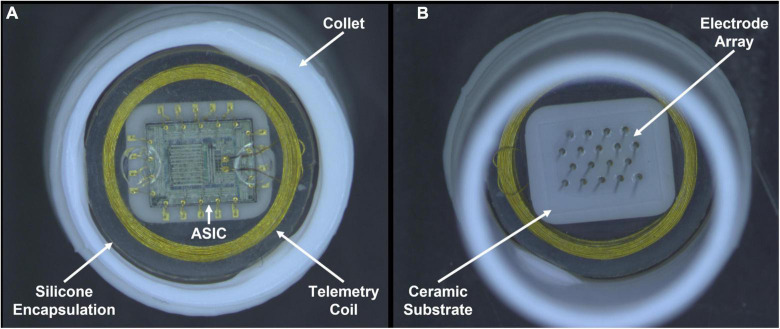
Photographs recorded after pulsing in LP-PBS for 24 days. One wireless floating microelectrode array (WFMA ID W0124_64_187) from the pulsed group of devices is shown inside a white collet used for device handling. **(A)** Backside of the WFMA viewed from underneath showing the gold wire telemetry coil, application-specific integrated circuit (ASIC), silicone encapsulation, and white collet used to contain each device during testing. **(B)** WFMA viewed from above showing the microelectrodes coming out of the ceramic substrate and silicone device encapsulation. No evidence of degradation was observed for the encapsulation, ASIC, or microelectrode array at the end of the 24-day pulsing study.

Devices were kept in their original collet and placed into two screw-top glass jars, one containing pulsed group devices and one containing unpulsed group devices. Figure 1 shows photographs of a WFMA device while inside its collet. 25 mL of test electrolyte (phosphate buffered saline with a low phosphate concentration, LP-PBS) was then added to each jar and the total mass of each test cell (glass jar and lid, four WFMAs in collets, and 25 mL LP-PBS) was recorded. The LP-PBS was allowed to air-equilibrate for the pulsing study, and contained salt concentrations of 126 mM NaCl, 5 mM Na_2_HPO_4_, and 1.4 mM NaH_2_PO_4_. LP-PBS has previously been used to characterize the effect of electrolyte composition on charge injection properties of AIROF electrodes (Cogan et al., 2007). The low phosphate buffer concentration, compared with the more typical PBS used to characterize the electrochemical properties of AIROF prior to *in vivo* studies (Troyk et al., 2007), produces a cyclic voltammetry response for iridium oxide that is closer to that observed *in vivo* compared with PBS (Kane et al., 2013). The jars with LP-PBS were maintained in a large, air-circulating oven at 37°C throughout the study. Slight water loss due to evaporation was measured by the change in mass of each jar from baseline and was compensated for with the addition of deionized water when necessary. The test electrolyte was agitated at least every 48 h to circulate solution and prevent dissolved gases from forming bubbles on or near the WFMA and collet. All WFMAs were soaked for a minimum of 12 h in LP-PBS at 37°C before recording initial measurements and initiating pulsing.

#### Saline pulsing protocol

The unpulsed control group was kept immersed in LP-PBS in the same oven as the pulsed group and no electrical stimulus was applied except as needed for periodic measurements of voltage transients. Pulsed WFMAs were subjected to accelerated current pulsing tests such that the total charge delivered through each electrode would be equivalent to the estimated maximum charge delivered through each electrode in a study subject over an anticipated 5-year period as part of a clinical trial of a vision prosthesis (NCT04634383). The estimated total charge that would be delivered by an individual electrode in the 5-year clinical trial is shown in Table 2 and is based on the approved psychophysical testing protocol for the trial.

**TABLE 2:** Calculated maximum charge for each electrode (Troyk et al., 2007; Kane et al., 2013).

Parameter	Value
Anticipated duration	5 years
Daily electrode use	4 h
Expected clinical testing	3 days/week 3 weeks/month
Clinical frequency	100 pulses/second
Duty cycle	50%
Total number of pulses	3.888 × 10^8^ pulses
Maximum current	80 μA
Clinical pulse width	200 μs
Maximum charge/Pulse	16 nC
Calculated total charge	6.22 Coulombs

**FIGURE 2:**
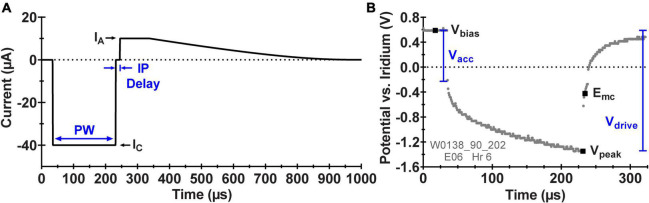
**(A)** Schematic diagram of the asymmetric, biphasic current waveform applied by the ASIC during pulsing with the WFMA labeled with the pulse width (PW), cathodic current amplitude (I_*C*_), anodic current amplitude (I_*A*_), and interphase delay (IP Delay). **(B)** Example *in vivo* voltage transient (VT) response to pulsing at 40 μA labeled with the set interpulse voltage bias (V_*bias*_), the most negative voltage excursion (V_peak_), and analysis parameters: maximum cathodic polarization potential (E_mc_), access voltage (V_acc_), and driving voltage (V_drive_).

Asymmetric, cathodal-first biphasic current pulsing, as shown in Figure 2A, was applied at a modestly accelerated rate of 200 pulses per second at the stimulator’s maximum cathodal current output of 80 μA and at the likely clinical-use pulse width of 200 μs (16 nC per pulse). Anodal recharge current was limited to 10 μA maximum amplitude, and the interphase delay was set at 12.4 μs. All WFMA electrodes for the long-term pulsing study had a geometric surface area of 3000 μm^2^, resulting in a maximum charge injection density of 533 μC/cm^2^. The stimulation parameters for the accelerated pulsing study are provided in Table 3. The maximum frequency for stimulation is 200 Hz, based on the results of prior studies showing changes to AIROF voltage transients when pulsed at higher frequencies (Cogan et al., 2004). Under accelerated test conditions (acceleration factor of 81), the study was condensed to a 23-day continuous pulsing period. An additional 1 day was added to the duration of the study to account for pauses in pulsing during voltage transient measurements.

**TABLE 3:** Accelerated pulsing parameters.

Parameter	Value
Duty cycle	100%, continuous study duration
Frequency	200 pulses/second
Current	80 μA
Pulse width	200 μs
Charge/Pulse	16 nC
Test duration	24 days (540 h)
Acceleration factor	81

#### Voltage transient measurements

After the initial 12-h equilibration period, baseline voltage transients (VTs) were recorded for all *n* = 8 WFMAs with 80 μA, 200 μs (16 nC/phase) cathodic current pulses applied at 200 Hz. All pulsing of the WFMA (for both stimulation and voltage transient measurements) occurred from an interpulse bias of +0.6 V versus each device’s uninsulated iridium reference electrode to provide charge-balance and to maximize charge-injection capacity (Troyk et al., 2004; Cogan et al., 2006). In saline with a pH 7.4, the equilibrium potential of an iridium metal electrode versus Ag|AgCl is approximately +200 mV.

After pulsing began, the voltage transient response was recorded every two days for both the pulsed and unpulsed groups starting on study day 2 and ending on day 24. VTs were recorded using the reverse telemetry feature of the WFMA system for which one voltage sample is transmitted per stimulation pulse, and a sequence of stimulation pulses is used with a time shift in each sample in order to create a composite VT waveform. A set of criteria were established for assessing if WFMAs should continue accelerated testing at each time point. (i) The magnitude of the driving voltage (V_drive_) must be less than 3.5 V, which is the maximum voltage supply for the device. If the magnitude of the transient tries to exceed 3.5 V, there will not be enough compliance voltage available to drive the desired current stimulus and the applied pulsing might not remain consistent over the remaining study duration. (ii) The maximum cathodic potential (E_mc_) of the electrode must be less negative than -0.6 V versus the iridium reference. That is, the AIROF electrode potential must remain more positive than the expected potential for the onset of water reduction (-0.6 V vs. Ag|AgCl in pH 7.1 phosphate buffered saline), which has been shown to lead to delamination of activated iridium oxide films (Troyk et al., 2004). An iridium electrode in saline will be approximately 200 mV positive of Ag|AgCl. Meaning the criteria selected for the study sets a conservative limit of approximately -0.4 V vs. Ag|AgCl. Figure 2B shows a representative VT from one electrode labeled with the parameters used for analysis and performance tracking over time: E_mc_, access voltage (V_acc_), and V_drive_.

#### Iridium dissolution measurements

Delamination of AIROF was shown *in vivo* during stimulation at 3 mC/cm^2^ (60 nC/phase, 100 μA, 600 μs pulse width) (Cogan et al., 2004), but it remains unknown at what combination(s) of pulse width, current amplitude, frequency, and duration of neural stimulation iridium metal dissolution may occur. And so, we evaluated dissolution of iridium metal during continuous pulsing in LP-PBS up to the estimated total charge delivered over a 5-year clinical trial (6.22 C at 16 nC/phase). Iridium metal dissolution was assessed throughout the study for both the pulsed and unpulsed groups using inductively coupled plasma mass spectrometry (ICP-MS). At baseline, 5 mL of electrolyte was removed from each test cell and placed into separate sealed glass vials. Samples were sent for ICP-MS analysis at Galbraith Laboratories (Knoxville, TN). The removed electrolyte was replenished with 5 mL of new LP-PBS solution added to each test cell. We continued to measure iridium dissolution throughout the pulsing study. For both pulsed and unpulsed array test cells, a 5 mL sample of the LP-PBS solution was removed at study days 6, 8, 12, 16, 20, and 24 and replaced in the same manner as baseline measurements.

#### End-of-study assessments

At the final study time point of day 24, VTs were recorded, and the final 5 mL electrolyte samples were collected for assessing iridium dissolution. All devices were moved to DI water for 24 h. The DI water was then replaced, and devices remained in DI water for an additional 24 h to ensure chemical species from the LP-PBS were thoroughly removed from within the AIROF. After soaking in DI water for a total of 48 h, devices were allowed to air dry at room temperature. Optical microscopy images were repeated for the *n* = 4 pulsed arrays and scanning electron microscopy (SEM) images were recorded for all *n* = 8 WFMAs (pulsed and unpulsed).

### Pulsing in canine visual cortex for 24 h

#### Device implantation

**FIGURE 3:**
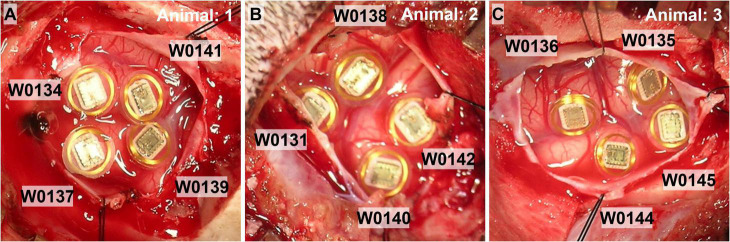
Photographs of the four WFMA devices after implantation in each of the n = 3 animal subjects. **(A)** Animal 1, IDs: W0134_3_198, W0137_58_201, W0139_65_203, and W0141_44_205. **(B)** Animal 2, IDs: W0131_52_195, W0138_90_202, W0140_12_204, and W0142_14_206. **(C)** Animal 3, IDs: W0135_7_199, W0136_113_200, W0144_97_208, and W0145_109_209.

The surgical procedure and stimulation study were performed at the University of Chicago Animal Resources Center. Three purpose-bred hounds were used for the study, and females were chosen because they have less mass in the temporalis muscle, and so it did not require dissection at the implant site to accomplish telemetry signal transmission. Animals were initially sedated with Buprenorphine (8.4 μg/kg). Hair over the scalp and neck was removed with clippers, IV lines were placed, and the animals were intubated. Animals were then placed in a Pavlovian-style surgical frame to secure the skull. Animals were initially given general anesthesia of ketamine (2.8 μg/kg) and dexmedetomidine (12.6 μg/kg), and then maintained on isoflurane (1.0–2.5%). WFMA’s used for the study had the same design specifications as those used in the LP-PBS pulsing study. Four WFMA’s were implanted in each of the *n* = 3 dogs, attempting to place each WFMA on the top of gyri in or near the right visual cortex as shown in Figure 3. The average implantation time (loading the insertion tool, implanting the device, documenting device identifier, photographing device location) for the WFMAs was about 4 min each. An electromechanical inserter tool was used to insert each WFMA at a rate of 1 m/s. Total time for the surgical procedure was just over 2 h. After implanting the four WFMAs, the dura and scalp were closed and each animal was allowed to recover from the implantation surgery for two weeks.

#### Stimulation protocol

The same asymmetric, cathodal-first biphasic current waveform that was used in the LP-PBS study was applied to WFMA electrodes in cortex. Two weeks after device implantation, continuous stimulation was applied for a period of 24 h. Two weeks post-implantation was chosen for running the 24 h stimulation study to allow a majority of the acute immune response to subside (Polikov et al., 2005), while minimizing potentially significant encapsulation around the electrodes that might mask the effect of stimulation on healthy neurons nearby.

Stimulation was applied to 8 electrodes within each of the four implanted WFMAs (out of 16 total electrodes) while the animals were under light anesthesia. Electrodes 1–8 were pulsed for seven of the implanted devices, and electrodes 9–16 were pulsed for the other five WFMAs. Selection of which WFMA device would be pulsed at which amplitude and what electrode group would be pulsed (1–8 or 9–16) was randomized. Table 4 summarizes the WFMA IDs for each animal subject, pulsed electrode groups, and pulsing amplitudes. Within each WFMA, pulsing was rotated among the 8 pulsed electrodes every 10 ms throughout the 24-h period so that stimulation was applied at only one electrode at any time. So, at all times there were four electrodes stimulating the visual cortex, one on each WFMA. Each WFMA was also pulsed at a different stimulation amplitude (cathodic phase): (1) at the maximum output of the WFMA, 79.9 μA (16 nC/phase), (2) at the anticipated clinical trial maximum, 63 μA (12.6 nC/phase), (3) at 40 μA (8 nC/phase), and (4) at 20 μA (4 nC/phase). All stimulation was applied at 100 Hz with a pulse width of 201.6 μs. During the sixth hour of stimulation, Dexamethasone 6.25 ml IV (1 ml/kg at 4.0 mg/min) was administered to each animal. Packed cell volume (PCV) *via* hematocrit tube centrifugation was measured to monitor and maintain fluid control. After the 24-h stimulation session, the animals were allowed to recover for two days and were examined for signs of visual or other neurological deficits. At the end of the two-day recovery period, animals were euthanized, and tissues prepared for later histopathological assessments.

**TABLE 4:** WFMA IDs and electrode group assignments for pulsing in dog.

Animal	WFMA ID	Electrodes	Amplitude
1	W0134_3_198	E01 – E08	80 μA
	W0141_44_205	E09 – E16	63 μA
	W0139_65_203	E09 – E16	40 μA
	W0137_58_201	E01 – E08	20 μA
2	W0142_14_206	E01 – E08	80 μA
	W0140_12_204	E01 – E08	63 μA
	W0131_52_195	E09 – E16	40 μA
	W0138_90_202	E01 – E08	20 μA
3	W0145_109_209	E01 – E08	80 μA
	W0135_7_199	E09 – E16	63 μA
	W0136_113_200	E01 – E08	40 μA
	W0144_97_208	E09 – E16	20 μA

#### Voltage transient measurements

The voltage transient response to applied constant-current pulsing was recorded periodically throughout the 24-h pulsing period using the reverse telemetry feature of the WFMA system. During the 24-h period, continuous stimulation was briefly paused during each round of voltage transient (VT) recordings. Initial (pre-implantation) VTs were recorded in phosphate buffered saline (PBS: pH 7.1–7.3; 0.126 M NaCl, 0.081 M Na_2_HPO_4_, 0.022 M NaH_2_PO_4_). VTs were repeated immediately after device implantation in cortex, then 2 weeks later at 0 h (before beginning the 24-h pulsing), 1 h, 6 h, 12 h, 18 h, 23.5 h, and 24 h (after ending the 24-h pulsing). Each VT was recorded in response to applying 39.9 μA (∼8 nC/phase), 202.3 μs pulses at 100 Hz.

### Data analysis and limitations of the wireless telemetry system

Each voltage transient recorded from the two studies was saved as a comma-separated-variable (CSV) file and the driving voltage (V_drive_), access voltage drop (V_acc_) and maximum cathodic electrode polarization (E_mc_) were determined during offline data analysis. E_mc_ was measured as the electrode potential at the termination of the cathodic current pulse, at the instant the current returns to zero before the interpulse delay. V_acc_ was measured as the electrode potential excursion at the start of the cathodic current pulse. V_drive_ is calculated as the difference between the most negative voltage excursion measured (V_peak_) and the interpulse bias (V_bias_, + 0.6 V vs. reference).

The methods used to accomplish wireless recording of voltage transients combined with the use of an iridium wire reference electrode results in a shift in the voltage values from the “true” potential that would be recorded at the electrode versus a Ag|AgCl or other more commonly used reference electrode for electrochemical measurements. Additionally, the voltage range over which the reverse telemetry system for the WFMA can record the transient waveform is smaller (∼3.0 V) than the range of the compliance supply for driving stimulation (∼3.5 V). And so, in some instances the VT waveform can appear as if the compliance voltage of the WFMA supply has been reached. This occurs when a large access voltage is present while driving higher currents, i.e., the expected VT curve flatlines (typically when applying currents above 40 μA *in vivo*). This behavior is a result of a limitation in the recording range available within the reverse telemetry system and does not necessarily correlate to exceeding the available compliance supply for driving stimulation. Due to this telemetry system limitation, we have chosen to exclude any VTs that “flatline,” or exceed the range of the voltage monitoring system, from our analysis. Ten out of 1,472 total VT measurements were excluded from the saline study data and 154 out of 1,728 total VT measurements were excluded from the canine study data. We do not expect exclusion of these VT data to significantly alter the results, as estimated values (based on V_acc_ at the beginning of the pulse) were generally within the standard deviation of non-excluded VT values.

## Results

### Stability in saline

#### Visual inspections

Optical microscopy did not reveal any differences in the integrity of devices before and after 24 days for either pulsed or unpulsed WFMAs. No differences were observed in the ASIC, telemetry coil, silicone encapsulation, electrode insulation, or AIROF films. We specifically looked for damaged wire bonds; separation of the elastomer from the ASIC, coil, or substrate; discoloration or presence of substances indicating corrosion on the coil, wires, or ASIC; AIROF delamination; and delamination of Parylene-C encapsulation. None of these features were present on any device, pulsed (Figure 1) or unpulsed.

**FIGURE 4:**
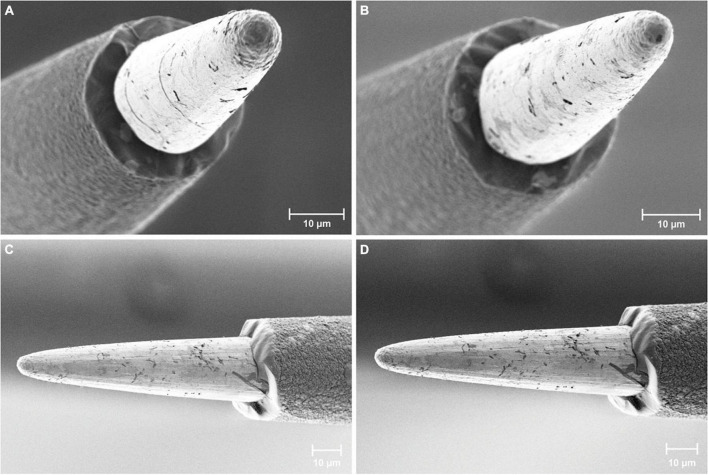
Scanning electron microscopy images of AIROF electrodes and Parylene-C encapsulation. Images show four different electrodes after 24 days in LP-PBS: two pulsed electrodes **(A)** E11 and **(B)** E12 from WFMA device W0089_63_152, and two unpulsed electrodes **(C)** and **(D)** from WFMA device W0132_101_196.

Scanning electron microscopy (SEM) investigations were used to assess the integrity of the AIROF electrode and Parylene-C encapsulation. No material defects were observed for any of the 16 electrodes in the WFMAs (*n* = 8). We did not observe any evidence of Parylene-C encapsulation opening at the junction between the AIROF and Parylene-C, nor was there evidence of exposure of the underlying iridium metal. Electrode tips retained their conical shape and there was no evidence of AIROF delamination. Some electrodes had residual Parylene-C that was not fully removed by the laser ablation process during fabrication. An example of 4 electrodes after the pulsing study is shown in Figure 4.

#### Iridium dissolution

No dissolved iridium was detected within the 0.05 μg/L sensitivity limit for the ICP-MS instrument for either the pulsed devices or controls with no pulsing. Therefore, we assumed that the maximum iridium dissolution was at the limit of detection, meaning the total iridium dissolution from the *n* = 4 pulsed WFMAs over the entire study period was less than 0.0025 μg.

Iridium metal or iridium ion toxicity levels in humans have not been determined. However, we have estimated that a maximum tolerable level of total iridium is 13.5 mg based on (i) a reported LD50 of iridium tetrachloride (58% iridium by weight) of 4.67 mg/kg in rat (Hazardous Substances Data Bank [HSDB], 2022), (ii) a factor of 1/10 to account for clinical use of up to 40 devices simultaneously (4.67 × 0.58 × 0.10 = 0.27 mg/kg), and (iii) assuming a minimum subject weight of 50 kg. The <0.0025 μg dissolution measured in this study is far below the estimated maximum allowable dissolution of 13.5 mg, and so we do not anticipate adverse reactions related to iridium dissolution or toxicity during *in vivo* use.

#### Activated iridium oxide film voltage transients in phosphate buffered saline with low phosphate concentration

**FIGURE 5:**
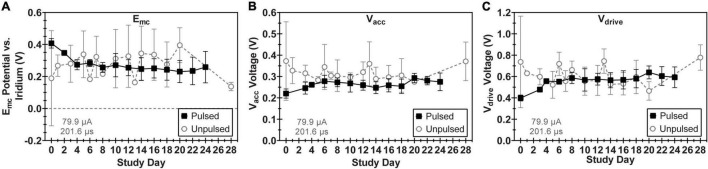
Comparison of voltage transient parameters for WFMA electrodes that were pulsed versus unpulsed throughout the 24-day study in LP-PBS. **(A)** Cathodic polarization potential (E_mc_), **(B)** access voltage (V_acc_), and **(C)** driving voltage (V_drive_). For the pulsed group, pulsing over a 24-day period was equivalent to 6.22 C of charge injection. Voltage transients were recorded in response to 79.9 μA, 201.6 μs pulses at 200 Hz.

All 16 electrodes pulsed in LP-PBS functioned normally throughout the pulsing period for each WFMA (*n* = 8) except for one electrode on an unpulsed WFMA (E14, W0132_101_196) that was not responsive from day 3 through the end of the 24-day study. No differences were observed between pulsed and unpulsed WFMA devices, although unpulsed devices had higher standard deviation values in all VT parameters (E_mc_, V_acc_, and V_drive_) than pulsed WFMAs. Figure 5A reports E_mc_ over the 24-day study period for pulsed and unpulsed electrodes in response to 79.9 μA, 201.6 μs pulses in LP-PBS (16 nC/phase, 0.8 mC/cm^2^). For all devices, the E_mc_ remained more positive of the -0.6 V potential limit criteria (versus the iridium reference) used throughout the study. The most negative E_mc_ observed on any electrode for all WFMAs was approximately -0.48 V vs. iridium (120 mV positive of the -0.6 V limit). E_mc_ values appeared to stabilize after day 10 of the pulsing study. Figure 5B compares the access voltage (V_acc_) for pulsed and unpulsed electrodes over the 24-day study period. The highest and lowest magnitude of V_acc_ in LP-PBS were approximately 0.94 V and 0.15 V, respectively. Figure 5C compares the driving voltage requirements for pulsed and unpulsed electrodes over the 24-day study period. For all *n* = 8 WFMAs tested, the driving voltage (V_drive_) required to deliver 16 nC/phase was less than the 3.5 V supply available within each WFMA device. The V_drive_ required to drive 79.9 μA current pulses also remained stable throughout the 24-day study period in LP-PBS. The higher standard deviation observed for unpulsed electrodes may be caused by variability in AIROF films after initial activation (through the wireless link). Applying stimulation to the films may actually increase uniformity of the oxide layer, as stimulation will cause slight changes in volume as reduced and oxidized species move in and out of the film. Thereby causing pulsed electrodes to have a lower standard deviation than unpulsed electrodes.

### Stability in canine visual cortex

**FIGURE 6:**
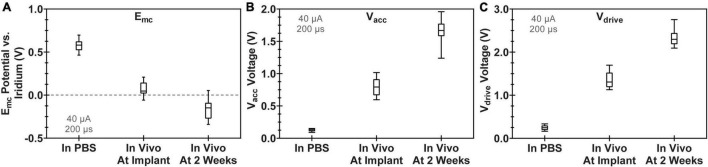
Comparison of voltage transient parameters for WFMA electrodes after manufacture (VTs recorded in PBS), at the time of device implantation, and 2 weeks after device implantation (before beginning the 24-h pulsing study). All VTs were recorded in response to 40 μA, 200 μs pulses at 100 Hz. **(A)** Cathodic polarization potential (E_mc_), **(B)** access voltage (V_acc_), and **(C)** driving voltage (V_drive_).

Voltage transient measurements confirmed the normal operation of all 12 WFMAs implanted in canine visual cortex. VTs also confirmed the expected delivery of current during stimulation for each electrode in the pulsed group. Figure 6 compares VT parameters measured in PBS with VT parameters in cortex at the time of device implantation and after 2 weeks *in vivo*, just before beginning the 24-h pulsing study. Data represent the average value across all electrodes and all 12 WFMAs used in the study. From the time of device implantation to the end of the 2-week recovery period, average E_mc_ became 224 mV more negative, average V_acc_ increased by 867 mV, and average V_drive_ increased by 976 mV.

**FIGURE 7:**
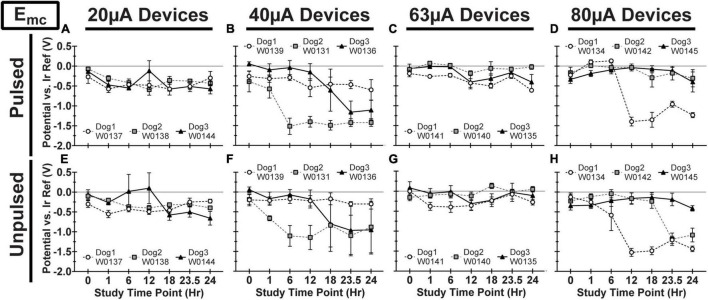
Cathodic polarization potential (E_mc_) vs. iridium reference electrode for pulsed **(A–D)** and unpulsed **(E–H)** electrodes in WFMA devices in the four different pulsing groups: 20 μA **(A)** and **(E)**, 40 μA **(B)** and **(F)**, 63 μA **(C)** and **(G)**, and 80 μA **(D)** and **(H)**. Voltage transients (VTs) were recorded at 0 h (before beginning the 24-h pulsing study), at 1h, 6h, 12h, 18h, and 23.5h (during the 24-h pulsing study), and at 24 h (after the 24-h pulsing study was complete). All VTs were recorded in response to 40 μA, 200 μs pulses. All data shown are mean and standard deviation across all electrodes within the group. All graph axes have the same scale.

**FIGURE 8:**
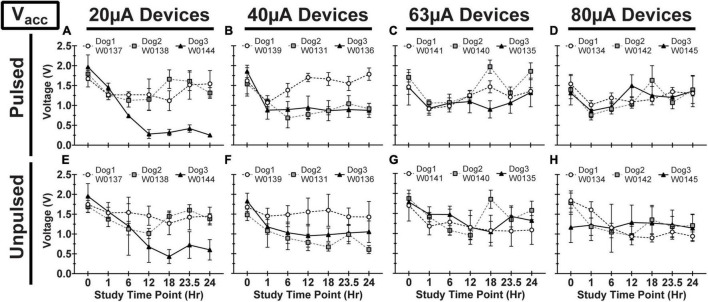
Access voltage (V_acc_) for pulsed **(A–D)** and unpulsed **(E–H)** electrodes in WFMA devices in the four different pulsing groups: 20μA **(A)** and **(E)**, 40 μA **(B)** and **(F)**, 63 μA **(C)** and **(G)**, and 80 μA **(D)** and **(H)**. Voltage transients (VTs) were recorded at 0hr (before beginning the 24-h pulsing study), at 1h, 6h, 12h, 18h, and 23.5h (during the 24-h pulsing study), and at 24 h (after the 24-h pulsing study was complete). All VTs were recorded in response to 40 μA, 200 μs pulses. All data shown are mean and standard deviation across all electrodes within the group.

**FIGURE 9:**
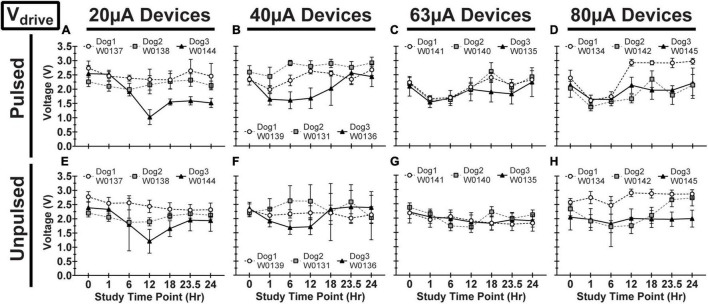
Driving voltage (V_drive_) for pulsed **(A–D)** and unpulsed **(E–H)** electrodes in WFMA devices in the four different pulsing groups: 20 μA **(A)** and **(E)**, 40 μA **(B)** and **(F)**, 63 μA **(C)** and **(G)**, and 80 μA **(D)** and **(H)**. Voltage transients (VTs) were recorded at 0hr (before beginning the 24-h pulsing study), at 1h, 6h, 12h, 18h, and 23.5h (during the 24-h pulsing study), and at 24h (after the 24-h pulsing study was complete). All VTs were recorded in response to 40 μA, 200 μs pulses. All data shown are mean and standard deviation across all electrodes within the group.

We observed variability in VT waveforms recorded *in vivo* across WFMAs and stimulation current levels, for both pulsed and unpulsed electrodes. In particular, V_acc_ and E_mc_ in some devices varied greatly over time during the 24-h pulsing study. The variability we observed *in vivo* was absent in VT data from our LP-PBS testing of the AIROF electrodes (Section “Stability in saline”) and was consistent with previously reported behavior of AIROF electrodes implanted in neural tissue (Cogan, 2006; Negi et al., 2010). The changes observed *in vivo* were not associated with any failure or reduced capacity to deliver charge for any electrode. VT data recorded after the end of the 24-h pulsing study indicated electrodes were still able to deliver stimulation up to 80 μA (16 nC/phase). Figures 7–9 summarize the net polarization (E_mc_), access voltage (V_acc_), and driving voltage (V_drive_), respectively, extracted from VT measurements of pulsed and unpulsed electrodes throughout the 24-h pulsing study. Data at 0 h were recorded on the day of the pulsing study (2 weeks after device implantation) immediately before beginning the pulsing period. Data at 24 h were recorded immediately after ending the 24-h pulsing period.

## Discussion

The durability of the WFMAs and stability of the AIROF electrodes pulsed in LP-PBS at 16 nC/phase (0.8 mC/cm^2^), within the recognized limitations of a non *in vivo* study, were notable. The assessment, based on voltage transients, optical microscopy, scanning electron microscopy, and iridium dissolution measurements over the 24-day pulsing period showed no evidence of degradation in AIROF charge-injection capability or progressive damage to the AIROF electrodes. Additionally, we did not measure any significant iridium dissolution when pulsing the AIROF electrodes for 24 days; however, it is possible there may have been some dissolved iridium present in LP-PBS samples in concentrations less than what we could measure with the ICP-MS instrument. We estimated that less than 0.025 μg of total accumulated iridium dissolution occurred from pulsed arrays over the 24-day pulsing period based on the detection limit of the instrument.

The study in saline, LP-PBS, involved four WFMAs subjected to pulsing at the 80 μA maximum current output of the WFMA stimulator (equivalent to 16 nC per phase), for the anticipated maximum number of pulses that would be employed over a 5-year clinical trial period (388.8 million pulses). The average AIROF polarization (E_mc_) was generally positive of the iridium reference electrode potential over the entire 24-day pulsing period, for pulsed and unpulsed controls, suggesting that the AIROF potential generally remains well-positive of the water reduction potential for AIROF (Beebe and Rose, 1988). Polarization of one electrode to -0.48 V versus the iridium reference was observed. Given that the iridium potential is approximately 0.2 V versus Ag|AgCl in saline at pH 7.2, polarization to -0.48 V vs. iridium is still about 0.32 V positive of the -0.6 V vs. Ag|AgCl water reduction potential. Likewise, the driving voltages (V_drive_) determined from VT measurements in LP-PBS indicate that the WFMAs can continuously supply up to 16 nC per pulse and remain within the device’s compliance limit. Since both E_mc_ and V_drive_ remained stable from day 10 through day 24, we expect a larger total number of pulses could be delivered than what was applied in this study without observing a change in electrode performance. Only one apparent electrode failure (E14, W0132_101_196) was observed in VT measurements. This failure occurred on day 3, and we suspect it was due to a WFMA manufacturing defect, possibly a wirebond failure or other defect in the ASIC, although we could not confirm this by microscopy. Overall, there was no evidence of WFMA corrosion seen in either optical microscopy or scanning electron microscopy images, or AIROF delamination in either pulsed or unpulsed devices.

The pulsing study in canine cortex was designed to allow two weeks of recovery post-implantation prior to initiating pulsing. This time interval was chosen to provide some time for acute implantation trauma to resolve and the tissue response to the implanted electrodes to partially stabilize, although tissue remodeling may continue for up to 6 weeks or longer after device implantation (Polikov et al., 2005). All three animals recovered without neurologic sequela and there was no evidence of reduced visual acuity during the observation periods after surgery and after stimulation. Observations of the WFMAs at the time of explant did not indicate the presence of migration or any dislodged devices that were either pushed out of or sunken into the superficial layers of the cortex. Even so, and not unexpectedly, there was considerable variability in the VT response of the pulsed and unpulsed AIROF electrodes. The 2 week recovery period before stimulation may have contributed to this variability; however, we expect 24 h continuous stimulation would cause distinguishable tissue damage at high levels of charge, at any time point post-implantation. The intent of the 2 week recovery period used here was to minimize immune activity but also avoid allowing significant fibrotic tissue encapsulation to occur.

Assessments of similar intrafascicular arrays with platinum microelectrodes showed a loss of functional stimulation electrodes when tracked for one-year post-implantation, where the maximum applied current was 100 μA (George et al., 2020). In this study, AIROF electrodes were subjected to continuous pulsing at either 20, 40, 63, or 80 μA for a period of 24-h, which corresponds to a total delivered charge for each current level of approximately 0.035 C (0.13 mC/cm^2^), 0.07 C (0.27 mC/cm^2^), 0.11 C (0.42 mC/cm^2^), and 0.14 C (0.53 mC/cm^2^), respectively. With respect to the stability and reversible charge-injection limits of positively biased AIROF determined in buffered saline, these levels of stimulus range remain within reported limits for avoiding water electrolysis (Beebe and Rose, 1988; Cogan, 2008; George et al., 2020). However, charge-injection limits determined *in vivo* using the same electrolysis-limit criterion for the maximum injectable charge are notably lower (Hu et al., 2006). Therefore, reported measurements from saline studies can only act as a guide in assessing the likely electrode damaging effect of the charge densities employed in the canine study. Although electrodes on each WFMA were pulsed at different charge levels, for comparison between WFMAs and electrodes, the condition of all electrodes was assessed from the voltage transient response to 40 μA pulsing. At this stimulus intensity, most electrodes remained stable throughout the 24-h study, with the largest changes typically occurring within the first hour of continuously applied stimulation.

In addition, it is interesting that electrodes within the same WFMA generally exhibit the same behavior over time. If a change was observed in E_mc_, V_acc_, or V_drive_ at a particular timepoint then the same trend was typically observed for all 16 electrodes in both the pulsed and unpulsed groups within the device. This suggests a physiological or environmental change at the implant site may have affected VT data during the study. Indeed, maintaining the physiological state of each animal when under anesthesia for an extensive time period of 24-h was very challenging. And so, we believe systemic changes or changes in the local environment near the electrodes over the course of the 24-h study could have caused shifts in the VT response on a whole-device level, explaining why both pulsed and unpulsed electrodes often exhibited the same changes in E_mc_ (W0131 and W0134), V_acc_ (W0144), or V_drive_ between VT recording time points. Even so, within the limits of the reverse telemetry feature of the WFMA, we were able to track changes in voltage transient parameters over the course of the 24-h pulsing period. As noted in Methods (Section “Data analysis and limitations of the wireless telemetry system”), there are limitations to the maximum electrode voltage that can be telemetered, but the data confirmed the functioning of the WFMA, confirmed whether each electrode was functioning, and provided an indication of the electrode polarization (E_mc_) with respect to the implanted iridium reference electrode.

The variability observed in the E_mc_, V_acc_, or V_drive_ data demonstrated the heterogeneity of the VT response of the AIROF when pulsed *in vivo*. For example, for Dog 2 electrodes pulsed at 63 μA were polarized to an average E_mc_ that was approximately 0 V versus iridium and comparatively unchanged over 24 h. This level of polarization would be considered non-damaging for AIROF based on saline studies (Hu et al., 2006). However, for Dog 1 at 63 μA, E_mc_ showed a general trend toward increasing polarization which reached approximately -0.6 V versus iridium which is close to the expected *in vivo* potential for water reduction. Overall, however, there was little difference in the VT response between pulsed and unpulsed controls on the same WFMA. This observation should not be interpreted to mean the AIROF electrodes were unchanged by pulsing, but it does suggest that the AIROF retained an ability to inject charge for all charge density levels used in the study. Our maximum charge density of 0.53 mC/cm^2^ is also well-below the level of approximately 3 mC/cm^2^ charge density that was identified previously to damage AIROF electrodes pulsed in cat cortex (Cogan et al., 2004).

There are limitations to this study. 24-day pulsing in LP-PBS suggests that AIROF is stable at clinically relevant levels of charge injection. However, there is accumulating evidence that charge-injection *in vivo* is accompanied by higher levels of polarization and reduced charge-injection capacities even after compensating for higher resistive voltage drops in tissue (Cogan et al., 2016). In the canine study, the E_mc_ is measured against an implanted iridium electrode which has an uncertain half-cell potential in the animal. In unpublished acute work in rat cortex, we find the rest potential of iridium to be within ∼50 mV of a Ag|AgCl reference potential and thus we can approximately correlate potentials measured against iridium in the animal with those reported against Ag|AgCl. However, this assumes that the acute and chronic rest potentials of iridium are similar. In addition, the continuous 24-h pulsing regiment is unlikely to be used clinically, and certainly not in a vision prosthesis. It is unknown how periods of quiescence will affect the electrochemical stability of the AIROF, although lower pulsing frequencies and duty cycle are recognized to reduce stimulation-induced tissue damage (Cogan et al., 2016). Also, the total charge delivered in the canine study is only a fraction of that likely to be encountered. Therefore, additional research is desirable to further assess stability and dissolution when using AIROF electrodes for longer periods of stimulation and whether there is an effect of the physiological environment on dissolution. Lastly, we have not reported detailed histological outcomes from the canine study. A separate publication addressing post-stimulation tissue response is in preparation.

## Conclusion

Wireless floating microelectrode arrays were subjected to accelerated pulsing for 24 days in saline and for 24 h in canine visual cortex. No significant AIROF polarization was observed and driving voltage values indicated the WFMA can continuously supply up to 16 nC per pulse during chronic stimulation. No evidence of device degradation or electrode failure or delamination was observed for the WFMA device components, Parylene-C electrode encapsulation, or AIROF electrodes after pulsing in saline for 24 days. Stability of the AIROF electrodes implanted in visual cortex was assessed by voltage transient measurements recorded through a wireless link using the reverse telemetry capabilities of the WFMA. Only three of the twelve implanted devices showed notable changes in polarization, access voltage, or driving voltage during the 24-h study period. Changes observed in 3 WFMAs may have been a result of the extended time under anesthesia and difficulties in maintaining the physiological state of animals over a 24-h period. Voltage transient recordings did not show any notable differences between pulsed and unpulsed electrodes for any of the twelve implanted WFMAs. Results of the accelerated pulsing studies in saline and *in vivo* indicate that activated iridium oxide film electrodes used in the WFMA have stable charge injection capabilities for continuous stimulation at least up to the 16 nC per pulse tested here. Additionally, the ability to acquire voltage transients using the reverse telemetry feature of the WFMA is useful for assessing the *in vivo* polarization and driving voltage of implanted electrodes and monitoring trends in electrode behavior.

## Data availability statement

The raw data supporting the conclusions of this article will be made available by the authors upon request, without undue reservation.

## Ethics statement

The animal study was reviewed and approved by the Institutional Animal Care and Use Committee (IACUC) at the University of Chicago.

## Author contributions

ES completed data collection and initial analysis for the saline pulsing study. VT was responsible for overseeing and performing device implantation procedures for the 24-h pulsing study in canine visual cortex, as well as monitoring animal welfare during the 2-week recovery period. AJ-I recorded all SEM images of WFMA devices. RF completed final data screening and analysis and preparation of the manuscript. PT and SC contributed equally to study design and final editing of the manuscript, discussion, and conclusions. All authors contributed to the article and approved the submitted version.
